# Rebuilding Earth’s first skeletal animals: the original morphology of *Corumbella* (Ediacaran, Brazil)

**DOI:** 10.1098/rsos.250206

**Published:** 2025-05-21

**Authors:** Bruno Becker-Kerber, Javier Ortega-Hernández, James D. Schiffbauer, Rudy Lerosey-Aubril, Lucas Verissimo Warren, Marcello Guimarães Simões, Lucas del Mouro, Cristiane Barbieri Rodella, Miguel Angelo Stipp Basei, Nathaly Lopes Archilha

**Affiliations:** ^1^ Department of Organismic & Evolutionary Biology and Museum of Comparative Zoology, Harvard University, Cambridge, MA, USA; ^2^ Geological Sciences, University of Missouri, Columbia, MO, USA; ^3^ Sao Paulo State University Julio de Mesquita Filho, São Paulo, Brazil; ^4^ São Paulo State University Institute of Biosciences, Botucatu, São Paulo, Brazil; ^5^ FIT - Flextronics Instituto de Tecnologia, Sorocaba, São Paulo, Brazil; ^6^ Brazilian Synchrotron Light Laboratory, Campinas, São Paulo, Brazil; ^7^ Universidade de São Paulo, São Paulo, Brazil

**Keywords:** tubular fossils, skeletogenesis, biomineralization, taphonomy

## Abstract

The evolutionary onset of animal biomineralization in the late Ediacaran (*ca* 555–538 Ma) is marked by the global appearance of enigmatic tubular fossils with unresolved phylogenetic relationships. Among these, *Corumbella werneri* from the Tamengo Formation (Corumbá Group, Brazil) has been variously interpreted as affiliated with cnidarians or bilaterians. Using synchrotron imaging and machine learning, we analysed new specimens of *C. werneri* to reconstruct their original skeletal organization. Our findings reveal that *Corumbella’*s tubes were originally conico-cylindrical. Large individuals of *Corumbella*, including less compacted specimens, and compression experiments with modern annelid tubes all indicate that previous reconstructions of a quadrate outline and midline features were misled by taphonomic artefacts. We also show that the wall of *Corumbella* is composed of a single layer of ring-shaped elements. Unlike the fourfold symmetry of scyphozoans or the complex cataphract-like structures of Cambrian bilaterians (e.g. halkieriids, tommotiids and wiwaxiids), *Corumbella* displays structural similarities with other late Ediacaran corumbellomorphs, such as *Costatubus*. These taxa exhibit a distinctive barrel-on-barrel tube construction, with modular elements stacked on each other rather than nested. Our findings redefine *Corumbella*’s morphology and phylogenetic affinities, contributing to a broader understanding of early biomineralizing metazoans and their ecological roles in the Ediacaran biosphere.

## 1. Introduction

An abundant and globally widespread biota of late Ediacaran biomineralizing animals marked the transition from a Precambrian world dominated by abiotic/microbial precipitation of calcium carbonate to a Phanerozoic-like environment, where animals and algae became key contributors to Earth’s carbonate factory [1,2]. These early biomineralizing metazoans are typically characterized by tubular or conical-tubular constructions, as exemplified by the well-known genera *Sinotubulites* and *Cloudina*, respectively [3,4]. Only a few biomineralizing taxa lack a tubular construction, such as *Namacalathus* [5]. A growing consensus regarding the bilaterian affinities of some of these tubular organisms, backed by the preservation of soft tissues interpreted as through-guts [6], suggests that the latest Ediacaran period saw the establishment of a global ‘worm-world’ fauna [7], with some tubicolous taxa persisting into the early Cambrian [8–10]. However, the biological affinities of most Ediacaran tubes remain elusive, and interpretations about the original morphology or composition of some taxa varies drastically among studies.

Given their temporal position, Ediacaran tubular organisms are often grouped as members of the Nama-type assemblage (e.g. [11,12]). *Cloudina* is the most iconic example [13], although other important and originally calcified tubes include *Sinotubulites* [3,14], *Multiconotubus* [15] and possibly *Costatubus* [16]. Other contemporaneous tubular taxa have been reported as being non-biomineralizing, or originally organic, such as *Conotubus* [17], *Saarina* [16] and *Zuunia* [18]. However, it is worth noting that the distinction between originally mineralized or organic may not be as straightforward as previously thought [18].

The Ediacaran tubular fossil *Corumbella* from late Ediacaran units in Brazil and Paraguay [19] has been regarded as originally organic [1,20] or biomineralized [21,22]. Unlike typical biomineralized taxa, these millimetric, regularly ringed tubes are typically preserved compressed, with their walls often still present as calcite remnants [23], despite being broken. This flattening poses challenges for reconstructing the original three-dimensional shape, which in the case of *Corumbella* has been variously reconstructed, from elongated pyramidal and quadrangular in section [21,22,24] to conico-cylindrical and rounded in section [23]. Despite this, most studies have agreed on a cnidarian scyphozoan affinity [20,21,24–28] until only very recently where affinities to scleritome-bearing bilaterians were proposed [22]. In this study, we reappraise the three-dimensional organization of *Corumbella* using new specimens from the late Ediacaran Tamengo Formation in Brazil. Analysis of little compacted specimens, microtomographic imaging and actualistic experiments on tube-forming metazoans indicate that the tube of *Corumbella* was originally conico-cylindrical and biomineralized.

## 2. Material and methods

### 2.1. Fossil provenance and measurements

The studied specimens were collected from the Sobramil quarry (Tamengo Formation, Corumbá Group, *ca* 541 Ma) in central-western Brazil (19°0'3.80" S–57°37'12.70" W). All specimens came from a silty-shale level in this quarry. Detailed geological context can be found in the electronic supplementary material. Specimens are deposited in the Zoological Collection of the Universidade Federal de Mato Grosso do Sul, under the acronym ZUFMS 1069−1152.

Biometric measurements, including tube length and width, were taken from over 200 *Corumbella* specimens, ranging from long, more intact individuals to short, fragmented ones. The specimens were subcategorized based on whether they had a uniseriate or a biseriate appearance (e.g. electronic supplementary material, figure S2). A single series (or row) of rings along the entire length of the tube is exposed in uniseriate specimens, whereas biseriate tubes are characterized by two series of rings separated by a longitudinal crease (the ‘midline’ of [21,22,24]). Rare specimens display transitions between the two types. These specimens were excluded from the biometric analysis, which aims to determine whether uniseriate and biseriate tubes exhibit different biometric characteristics.

For all the other specimens, the total length and maximum width of the tubes, and the maximum height and thickness of the rings were measured (electronic supplementary material, figure S2). Ring height was only measured in fully exposed rings to limit the impact of partial imbrication on these measurements. The total length values should be considered as approximations of the original tube length since the fossils are either fragmented, or their extremities are often not visible due to the common conchoidal splitting of the shale or presence of fractures, which often terminate long tubes at the limits of the exposed surfaces.

### 2.2. Mechanical tests of modern skeletal tubes

We performed compression experiments with a mechanical press on modern extant sabellidan annelids sourced from the Invertebrate Zoology collection at the Museum of Comparative Zoology, Harvard University (Cambridge, MA) to better constrain patterns of breakage, collapse and deformation of organic and biomineralized tubes when submitted to uniaxial compression. The sabellidan taxa were selected because they possess tubes that are comparable in size and thickness to those of *Corumbella* while varying in composition. The organic tubes of *Escarpia* sp. (family Siboglinidae), the thick-walled biomineralized (aragonitic-calcitic) tubes of *Hydroides dianthus*, and the thin-walled biomineralized (aragonitic-calcitic) tubes of *Hydroides elegans* (family Serpulidae) were inserted between rectangular pieces of rubber sheets and then subjected to weak uniaxial compression by hand using a glass petri dish (*Escarpia* and *H. elegans*) or strong uniaxial compression using a mechanical press (*Escarpia* and *H. dianthus*). The manual press machine (VEVOR) used has an effective working area of 305 × 220 mm and can produce up to *ca* 220 kPa. The biomineralized tubes were not subjected to further compression upon collapse and fracturing. The tubes of *Escarpia* were compressed for a total of 30 min and the appearance of fractures was monitored periodically. The exact pressure at the moment of collapse and fracturing cannot be measured with the manual press machine.

### 2.3. Synchrotron radiation microtomography and machine-learning image processing

Four fossil samples were imaged using the Mogno Beamline at the SIRIUS synchrotron (Campinas, Brazil) and one sample was imaged using the DIAD beamline at the Diamond Light Source (Didcot, UK). The samples selected for microtomography were trimmed using a Dremel 4000 to remove excess surrounding host rock and then mounted either inside small glass funnels (DIAD) or directly on top of the sample holder. At the DIAD beamline (Diamond Light Source) the sample was imaged in 180°, using an unfiltered pink beam, a pco.edge 5.5 detector (sCMOS), effective pixel size of 0.5 µm and 5100 projections. Images were reconstructed using the filtered back projection (FBP) algorithm available in the Savu Python package. At the Mogno beamline, samples were imaged in the nanostation, with a 360° rotation angle, 22 keV quasi-monochromatic conebeam, variable sample-detector distance (depending on sample size), a pco.edge detector and 2048 projections. The effective pixel size varied between 0.5 and 2.8 µm. The tomographic images were reconstructed using in-house developed Python pipelines together with Astra and Tomopy functionalities.

The best microtomography dataset was selected for training a machine-learning model for image segmentation through the Annotat3D software [29]. The pipeline used was composed of: (i) subtracting the background (air) using Avizo; (ii) selecting a subvolume containing all the relevant features; (iii) annotating the features in Annotat3D and training the model across the entire subvolume; (iv) making necessary corrections to the annotations; (v) using Avizo for finer corrections of the machine-learning label output, finally creating a ‘gold standard’; (vi) using the gold standard label image along with the original subvolume to train the deep learning model; (vii) deploying the trained model to segment the full original microtomography dataset; and (viii) applying final label adjustments and rendering the segmented dataset in Avizo.

## 3. Results

### 3.1. General morphology

#### 3.1.1. Measurements

**Figure 1.**
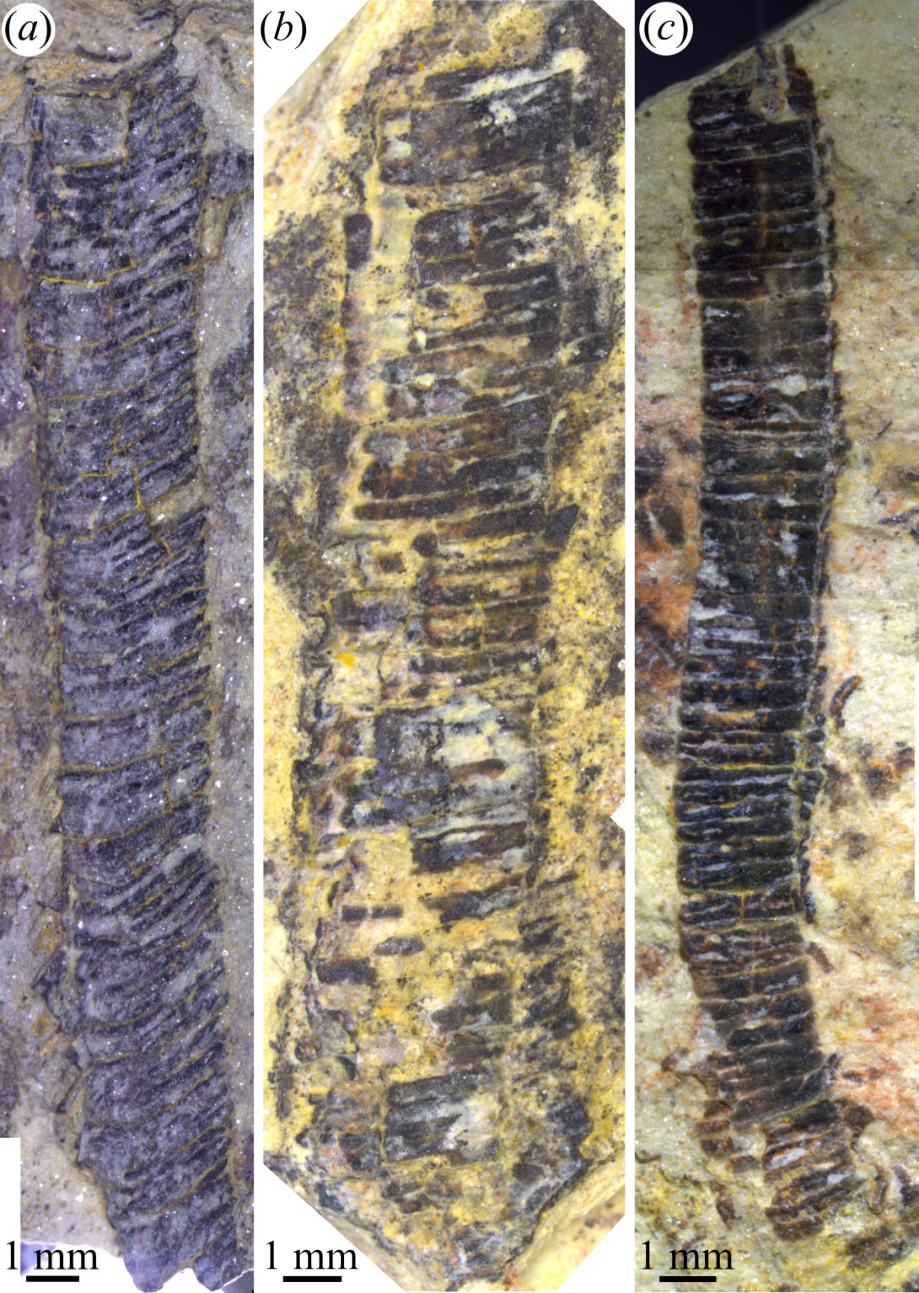
*Corumbella* specimens showing the wide and long uniseriate morphotype. (*a*) Sample no. ZUFMS-FOS01069. (*b*) Sample no. ZUFMS-FOS01070. (*c*) Sample no. ZUFMS-FOS01071.

**Figure 2.**
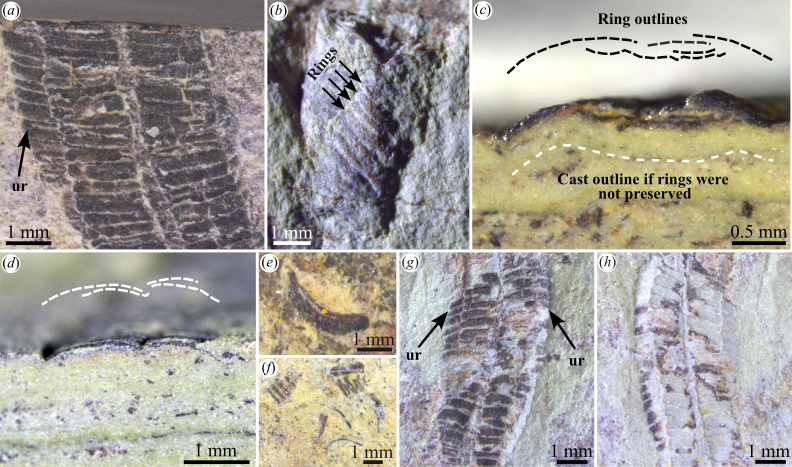
Ring superposition and morphology in *Corumbella*. (*a*) Specimen showing a seemingly additional row created by lateral displacement of underlying rings (ur); ZUFMS-FOS01072. (*b*) Specimen with preserved curvilinear to flattened semicircular shape; ZUFMS-FOS01073. (*c*,*d*) Transverse section of the specimen showing ring morphology; ZUFMS-FOS01074. (*e*,*f*) Reworked rings showing flattened arch morphology, or curvilinear outlines; ZUFMS-FOS01070, ZUFMS-FOS01075. (*g*,*h*) Laterally displaced underlying rings (ur) exposed on both sides of overlying rings (*g*), creating a polygonal cast in the counterpart (*h*); ZUFMS-FOS01076.

**Figure 3.**
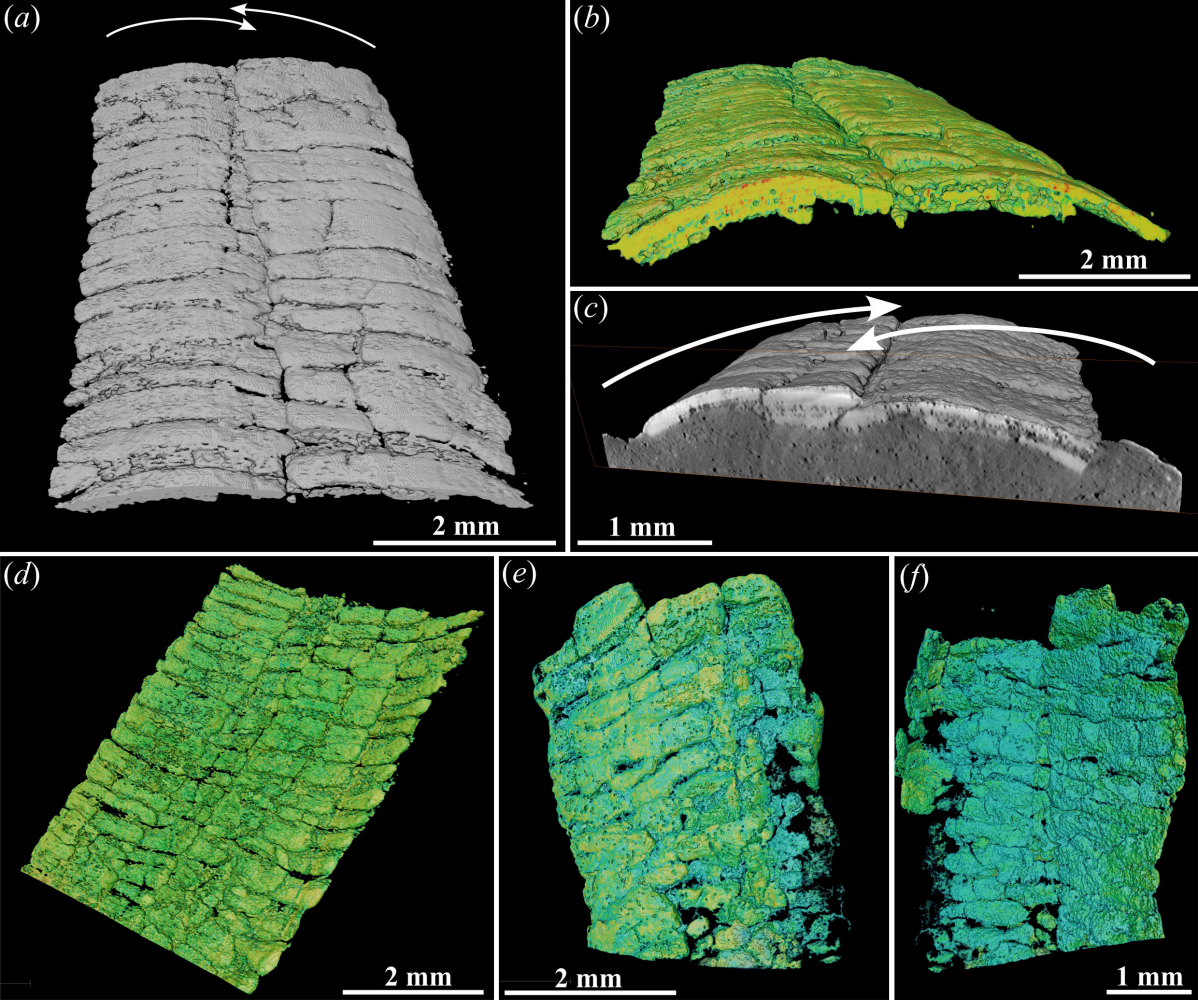
Synchrotron radiation micro-computed tomography (SR-micro-CT) images of *Corumbella*. (*a–c*) Rendered three-dimensional image of the exposed wall layer of a biseriate tube showing a partial overlap of ring series in the central region; ZUFMS-FOS01077. (*d*) Internal view of the specimen in (*a–c*) highlighting the presence of only two layers of rings. (*e*,*f*) Specimen with an exposed uniseriate wall (*e*), and an underlying biseriate one (*f*); ZUFMS-FOS01078.

*Corumbella* tubes on bedding surfaces occur in two morphotypes, uniseriate (figure 1, electronic supplementary material, figures S2, S3B and S4a,d) or biseriate (figures 2*a*,*c*,*d*,*g* and 3*a*–*d*, electronic electronic supplementary material, figures S3a and S4b,e,f), based on the absence or presence of a longitudinal crease, respectively. The width of biseriate specimens is normally distributed (Shapiro–Wilk W = 0.98; *p* = 0.75) and varies from 1.1 to 5.9 mm (*n* = 68; mean = 3.2 mm; electronic supplementary material, figure S3b,c and table S1). Uniseriate specimens are smaller on average, showing widths ranging from 0.5 to 5.2 mm (*n* = 143; mean = 1.7 mm), but are non-normal (Shapiro–Wilk W = 0.89; *p* < 0.001) and possibly show a multimodal distribution (electronic supplementary material, figure S3b and table S1). Wider uniseriate (greater than 3 mm) specimens are outliers (electronic supplementary material, figures S3c and table S1), with widths close to the maximum values observed for the biseriate tubes.

Tube length is highly variable between morphotypes, from 5.5 to 130 mm (mean = 26 mm) in biseriate specimens and from 0.4 to 37.8 mm in uniseriate specimens (mean = 3.6 mm). Most length values for the uniseriate specimens are below approximately 8 mm (electronic supplementary material, figure S3e). However, very long uniseriate specimens occur (figure 1), as can be noted in the length and length/width ratio boxplots (electronic supplementary material, figure S3e,f), with some fossils showing lengths up to 13 times the maximum width. Some of these long individuals (figure 1*a*,*b*) also have large widths of over 3 mm. There is no correlation between the maximum width and length of the specimens, which probably results from the fragmentary nature of the material or their uneven exposure of bedding surfaces. Minimal variation in ring height and thickness was observed within individual specimens. However, across the studied material, ring height ranged from 150 to 350 µm (mean = 240 µm), and ring thickness ranged from 22 to 112 µm (mean = 55 µm). Ring dimensions show no correlation with tube dimensions.

#### 3.1.2. Taphonomic variants

A few tube fragments show transitions between uniseriate and biseriate (and *vice versa*) along their length, a transition that is not associated with a significant change in tube width (electronic supplementary material, figure S4c,d). In specimens allowing adapical and adapertural regions to be determined based either on changes in width, or by very small widths (less than 1 mm) suggesting adapical portions, it can be shown that uniseriation and biseriation are not restricted to a specific region of the tube (electronic supplementary material, figure S4a–d).

Long tubes can also show shorter, apparently broken ring series that are slightly detached from the main tube axis (electronic supplementary material, figure S4e), and may seldom be found associated with shorter fragments (electronic supplementary material, figure S4f). Additionally, some slabs preserve shell hashes consisting of similarly sized tube fragments typically found clustered on surfaces that generally do not contain long specimens (electronic supplementary material, figure S4g).

The studied material primarily consists of compressed specimens (figure 2*a*), except for some fragments that retain a more three-dimensional curvilinear to flattened semicircular shape (figure 2*b*, electronic supplementary material, figure S5). In all the others, the exposed wall layer is in close association with a second wall layer it overlays, which indicates a collapse of the original structure typically with minimal sediment infill (figure 2*a*,*c*,*d*). The rings in transverse sections (figure 2*c*,*d*), and rings and tubes in perpendicular views (figures 2*e,f* and 3*b,c*), generally exhibit smooth, curvilinear shapes resembling flattened arches rather than sharp angles. On the surface, no tube consistently displays more than two series of rings along its length, but some exhibit three or even four series locally (figure 2*a*,*g*,*h*). These additional series arise from partial exposure of underlying rings (arrows in figure 2*a*,*g*). When present, such partially exposed rings produce moulds with polygonal shapes and angular ‘lateral edges’ in the counterparts (figure 2*h*).

Biseriate specimens are characterized by a ‘zigzag’ (or offset) disposition formed at the ‘midline’ (electronic supplementary material, figures S2 and S3a), although straight sets can occur as well (electronic supplementary material, figure S6e). A few rings may be continuous over the central region, locally interrupting the bipartite pattern and showing evidence of plastic deformation (electronic supplementary material, figure S6a–d). Rings can sometimes be displaced under adjacent units (arrow in electronic supplementary material, figure S6e).

#### 3.1.3. Three-dimensional characterization of *Corumbella*

**Figure 4.**
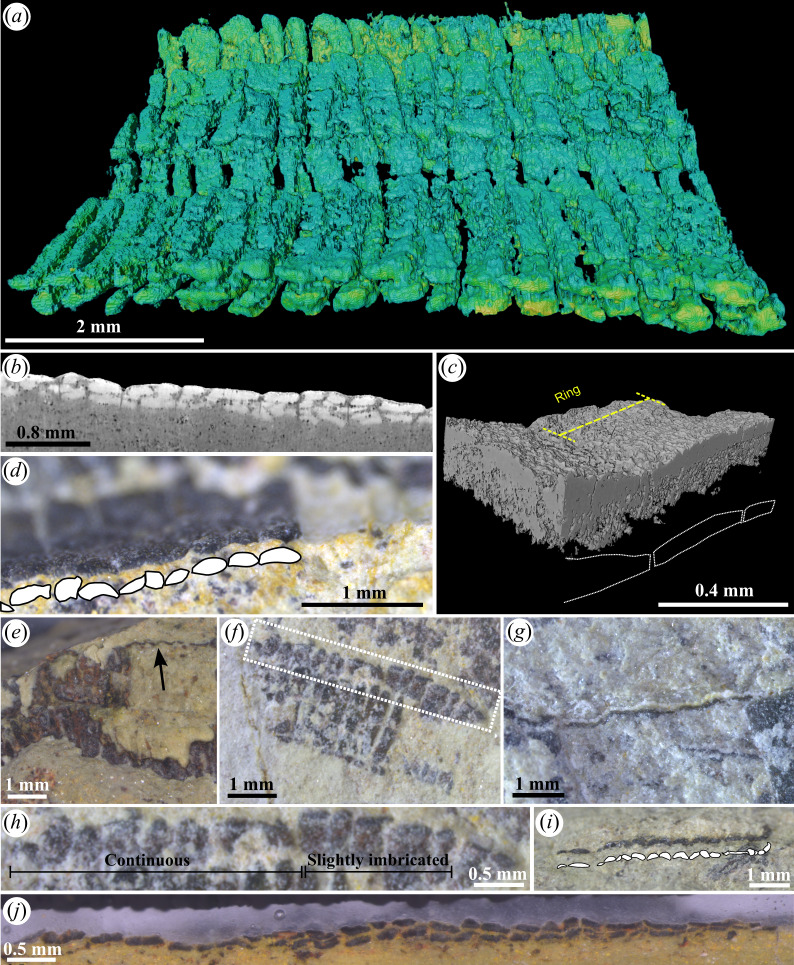
Ring morphology and disposition. (*a*) Rendered SR-micro-CT image of a flattened tube in longitudinal view, showing its two wall layers and evidence of alteration of their surfaces; ZUFMS-FOS01077. (*b*) Virtual longitudinal section of the tube above showing the ellipsoidal to allantoid shape of the rings in cross-section; ZUFMS-FOS01077. (*c*) Rendered SR-nanoCT image illustrating the non-imbrication of the rings composing the wall layer; ZUFMS-FOS01079. (*d*) Wall layer showing limited imbrication of some of the rings; ZUFMS-FOS01080. (*e*,*f*) Specimens with partially exposed underlying wall layers, showing the matching disposition of the rings on both wall layers; ZUFMS-FOS01081, ZUFMS-FOS01082. (*g*) Continuous rings with no imbrication in a vertically oriented wall at the lateral part of a tube; ZUFMS-FOS01083. (*h*) Specimen showing localized limited ring imbrication; ZUFMS-FOS01082. (*i*) Longitudinal section of a tube fragment illustrating the ellipsoidal, crescentic and elongated ellipsoidal shapes of its rings in cross-section; ZUFMS-FOS01084. (*j*) Polished longitudinal section of a tube showing the two wall layers and the ellipsoidal, more rarely sigmoidal shapes of its rings in cross-section; ZUFMS-FOS01085.

Transverse sections and synchrotron radiation micro-computed tomography (SR-micro-CT) images show that the two ring series composing one wall layer of a biseriate tube overlap in the central part (figure 3*a*–*d*; electronic supplementary material, videos S1–S3). Microtomography of large tubes reveals that an exposed uniseriate wall layer may overlay a biseriate one (figure 3*e*,*f*). Virtual (SR-micro-CT images) and polished longitudinal sections of *Corumbella* specimens show predominantly two superposed wall layers (figures 3*b–d* and 4*a,b,j*, electronic supplementary material, figure S7). Locally, and often towards the centre of the tubes, more layers can be observed due to the partial overlap of the two-ring series of each wall layer (figure 4*b*). Some tubes can also present only one wall layer, particularly at their lateral-most regions where there is no or little superposition and the rings are slightly vertical with respect to the bedding plane (figure 4*e*–*h*). Horizontal displacement (with respect to bedding plane) and partial dissolution of rings, can also explain the presence of more than two layers in sections (figure 4*b*, electronic supplementary material, figures S6e and S7c–e).

Study of bedding surfaces (figure 4*d*–*i*), polished sections (figure 4*j*) and micro-CT images (figures 3*d* and 4*a*, electronic supplementary material, figure S7a–c,e) show that the rings are usually arranged linearly, with little (figure 4*g*,*i*) to no contact between each other (figure 4*a*,*b*,*f*,*h*,*j*), as if they were separated skeletal elements. Locally in some specimens, the rings can also appear completely connected to adjacent units, which can be the result of recrystallization (figure 4*b*, electronic supplementary material, figure S8a). In any case, imbrication of the rings of a given wall layer is seldom observed and always limited in depth and localized, never extending to the full length of the tube (figure 4).

Longitudinal sections and views of tubes and vertically oriented rings show that the rings are generally elongated ellipsoidal in cross-section (figure 4; electronic supplementary material, video S1), but some may be slightly crescentic instead (e.g. figure 4*c*), or sigmoidal (some rings in figure 4*j*). Additionally, some rings occasionally appear as thin stripes. The surface of the rings is often rugged (figures 3 and 4*a,c*, electronic supplementary material, figure S7c,d).

### 3.2. Actualistic experiments

Uniaxial compression (i.e. equal and opposite forces on opposite sides of the tubes) of modern annelid tubes creates a midline fracture along the tube length, independently of whether the composition is entirely organic (*Escarpia* sp.) or biomineralized (*H. dianthus* and *H. elegans*) (electronic supplementary material, figure S8). The collapsing of the tubes in all cases also results from the development of lateral fractures.

The thin-walled (*ca* 14 µm) organic tubes of *Escarpia* sp. do not break using hand-pressed dishes, but plastically deform (electronic supplementary material, figure S8a). A central fracture only appears after 30 min of consistent pressure using a mechanical press (electronic supplementary material, figure S8b). This fracture does not run along the entire length of the tube, but it replicates its sinuous shape. The fracture edges are ragged, and the wall layer appears flattened and curvilinear in the transverse view (electronic supplementary material, figure S8f). Rare transverse fractures may also occur (electronic supplementary material, figure S8b).

The relatively thin-walled (*ca* 63 µm) biomineralized tubes of *H. elegans* fracture and collapse under even the slightest pressure by hand with the petri dish (electronic supplementary material, figure S8a). As in *Escarpia*, the central fracture follows the sinuous shape of the tube, even when the tube is adjacent and slightly overlapping other tubes, and it may be accompanied with rare transverse fractures (electronic electronic supplementary material, figure S8c,d). In transverse views, some wall fragments appear flattened and curvilinear (electronic supplementary material, figure S8g), while others retain a higher degree of curvature.

The thicker walled (*ca* 147 µm) biomineralized tubes of *H. dianthus* similarly exhibit a brittle behaviour, but they require a strong uniaxial compression with a mechanical press to fracture and collapse. Additionally, the central fracture co-occurs with many transverse fractures (electronic supplementary material, figure S8e), and the resulting fragments do not appear flattened in transverse view (electronic supplementary material, figure S8h).

## 4. Discussion

### 4.1. Tube (re)construction

The reconstruction of *Corumbella*’s original morphology has significantly evolved since its initial description over four decades ago as a uniseriate ‘primary polypar’ (interpreted as a stalk) transitioning to a biseriate region composed of ‘secondary polypars’ [25]. Subsequent studies have proposed either an elongate pyramidal geometry [21,24,30], or a cylindrical morphology similar to Ediacaran sinotubulitids [23]. According to the pyramidal model [21,30], the original cross-sectional shape of the tube transitions from circular near the apex to quadrangular toward the aperture. The wall was also later interpreted as consisting of two layers of imbricated ring units in a cataphract arrangement [22], alluding to the organization of sclerites in Cambrian-aged bilaterians, such as halkieriids and tommotiids. We find no evidence of an original quadrangular cross-section of the tube of *Corumbella* in our new material from the Tamengo Formation and argue that the pyramidal model and ‘midlines’ are based on a misinterpretation of taphonomic variants.

#### 4.1.1. Angular folds and three-dimensional facetted moulds

The presence of angular edges separating planar faces was inferred from two observations: the presence of incompletely flattened (arguably original) angular edges on collapsed tubes (figs. 5d–h and 8b–e in [21]), mostly preserved as moulds, and the partial preservation of a polyhedral three-dimensional morphology in moulds (figs. 2h and 8f in [21]). Transverse sections (figure 2*c*,*d*) and views of the tubes in transverse orientation (figure 3*b*,*c*, electronic supplementary material, figure S5a), along with rings oriented perpendicular or slightly inclined to the bedding plane (figure 2*e*,*f*, electronic supplementary material, figure S5b,c), consistently show that the ring fragments are curvilinear, with no evidence of lateral edges or corners. This indicates that the occasional angular folds are taphonomic in origin and probably result from brittle or plastic deformation of the tube during its compaction.

The presence of curvilinear rings in the same specimens that exhibit angular folds in previously published material further supports a taphonomic origin for the lateral edges (fig. 8b in [21]). Indeed, it is unclear how uniaxial compression (as in overburden pressure) would transform planar faces into curvilinear fragments (figures 2*b–f* and 3*b,c*, electronic supplementary material, figure S5). Additionally, and perhaps more importantly, collapsed tubes with the underlying wall layer jutting out from the sides of the exposed wall layer produce moulds that mimic a partial three-dimensional preservation of originally polyhedral tubes (figure 2*g*,*h*). This observation explains why a quadrangular cross-section is often apparent in moulds of the tubes, which includes most of the original material of *Corumbella* (see plates 1−3 in [25]) and specimens previously illustrated with ‘lateral edges’, (figs. 5d–h and 8c,f in [21], and possibly fig. 8b,d,e in [21]). It is important to note that some moulds of *Corumbella* after the dissolution of the walls usually have reddish to dark colours due to the presence of oxides [23], which can be mistaken as preserved walls. Significantly, less compacted specimens show a more semicircular outline reminiscent of an original cylindrical geometry (figure 2*b*, electronic supplementary material, figure S5a), which further refutes the four-sided wall hypothesis.

#### 4.1.2. Uniseriate and biseriate are not specific to tube ontogeny

The new material demonstrates that the medial crease of *Corumbella*, the defining character of the biseriate tubes, is inconsistently expressed, even in extremely wide and/or long tubes (figure 1). Uniseriate specimens can sometimes exceed the median and mean width of biseriate specimens (electronic supplementary material, figure S3c). For instance, some uniseriate examples reach sizes of 5.16 mm (electronic supplementary material, dataset S1), approaching the maximum width observed in biseriate tubes (5.86 mm). It can be concluded then that these large uniseriate specimens do not correspond to apically located circular regions of putative polyhedral tubes, as previously suggested [22] for small fragments figured in [23]. Although there are cases where the uniseriate fragments probably represent reworked broken ring series (electronic supplementary material, figure S3g), as they compare with the half-width of the biseriate tubes (electronic supplementary material, figure S3b,c), and as shown in short broken ring series that were just beginning to detach from long tubes (electronic supplementary material, figure S4e). However, all these cases represent small fragments with very short lengths (electronic supplementary material, figure S4e–g). This effect is clearly demonstrated in the length probability density function (electronic supplementary material, figure S3d), which suggests a rapid reduction in length due to the exposure of the tubes of dead individuals to taphonomic processes at the water/sediment interface, with occasional lateral transport.

This common, marked shortening of the tubes was facilitated by the loose (or absent) connection between the ring units (see figure 4*a*,*b*,*d*,*f*,*j*). The exceptions to this are the uniseriate tubes with large widths and lengths, producing the polymodal size distribution of the uniseriate type, and the outliers in the boxplots (electronic supplementary material, figure S3). These large uniseriate tubes also reinforce the interpretation of a circular symmetry over the fourfold configuration since they lack planar faces and borders. Together with the rapid shortening of the reworked fragments, this challenges the fragmentation model of *Corumbella* proposed previously (fig. 1f–I–II in [31]). Most short uniseriate fragments, which are about half the width of biseriate tubes, also contradict this model [22,31], especially since, under that framework, each uniseriate fragment would represent a complete articulated element (or ‘plate’ *sensu* [22,31]). If this were the case, compression of these units should yield widths consistent with the full tube, as each would include two half-faces in the pyramidal interpretation (see fig. 1–II–IV in [31]). Uniseriate tubes are also preserved in poorly compacted and recrystallized grainstone in Tagatiya Guazu Formation, Paraguay [20,32].

In sum, we find no evidence of a modification of the tube’s morphology towards the aperture. Even adapical regions can be biseriate due to compaction (electronic supplementary material, figure S4b), and conversely, apertural regions can be uniseriate (electronic supplementary material, figure S4d). These observations indicate that uniseriate portions are not restricted to a specific region of the tube in *Corumbella*. Specimens showing changes from uniseriate to biseriate patterns are found in three discrete scenarios: (i) forming from non-continuous breakage, with no observable change in width; (ii) forming from an apparent transition in specimens that are partly covered by sediments (electronic supplementary material, fig. S1B in [22]); or (iii) forming from an apparent transition, but with the broken series removed during reworking. In the latter cases, a more abrupt change in width is observed. Considering all the above, we interpret the biseriate patterns as not reflecting the original morphology and propose that they should be regarded as taphomorphs (see §4.1.3).

#### 4.1.3. The midlines are compactional cracks that can be reproduced experimentally

The midline is not an original anatomical feature, but a crack resulting from compaction. This is indicated by the fact that the midline is occasionally interrupted by rings that extend continuously from one lateral side of the tube to the other (electronic supplementary material, figure S6a–d). A compactional origin for the midline also explains the common observation of a partial overlap medially of the two ‘ring’ series (figure 3*a*–*c*), a feature expected when a cylindrical structure collapses. Fractures along the midline, as well as smaller breaks elsewhere in the tubes, are infilled with sediment, causing moulds of *Corumbella* to appear as though they contain internal septa (figure 2*h*). This accounts for the light-coloured ‘septa’ seen in previously illustrated specimens [21] and explains why some tubes exhibit ‘midlines’ that protrude externally.

Critically, we were able to experimentally produce compactional cracks on organic and biomineralized modern annelid tubes, which are similar in position (approximately equidistant from the lateral sides of the compressed tube) and path (running roughly parallel to these edges) to the midlines of biseriate tube fragments of *Corumbella* (electronic supplementary material, figure S8). This was in addition to the cracks along the lateral borders of the tubes, which also occur in the fossils. Our taphonomic experiments on annelid tubes demonstrate that the lateral-most and central regions of a cylindrical tube are the most vulnerable to rupture, regardless of the composition and thickness of the wall. Although this approach does not replicate the exact conditions during the entire compaction history of sedimentary successions, it can provide information on the breakage patterns of the tubes after collapse.

This type of fracture could be informative with regard to the *Corumbella’*s wall composition. The most similar breakage pattern observed was in the relatively thin-walled, biomineralized tubes of *H. elegans*. Given the similar wall thickness in both taxa (*ca* 80 µm), this comparable breakage pattern may be interpreted as supporting evidence of biomineralization in the *Corumbella* tube. Analogous patterns of deformation in artificial cylindrical tubes were also observed before [23,33]. It is also worth noting that a central collapse is commonly observed in other Ediacaran/Cambrian tubular fossils, such as *Costatubus* [16], *Hyolithellus* [34] and *Byronia* [35], with potential implications for their morphological interpretation. Finally, compression experiments [36–38] using artificial square tubes produce deformation patterns that differ markedly from those observed in cylindrical geometries, but match what is observed in conulariids (e.g. [39]), an extinct group with well-known pyramidal geometry.

#### 4.1.4. Structure of the wall

We argue that the apparent bi-layered wall observed on longitudinal sections by previous authors [22] simply represents the wall layers corresponding to the two opposite sides of the compressed tube. The *Corumbella* tubes observed in virtual longitudinal views, physically polished sections and transverse views predominantly show only two wall layers (figures 3*b,c* and 4*a,b,j*, electronic supplementary material, figure S8). If the walls were bi-layered and the rings composing it were imbricated, its compaction should lead to four or more wall layers being visible along most of the tube length in sectioned views, which is not the case here (figures 3*b,c* and 4*a,b,j*, electronic supplementary material, figure S8e and videos S1–S3) or in previously published [22] micro-CT datasets (electronic supplementary material, figure S8a–d). Three to four wall layers may be locally observed in some specimens, and this can be explained by superposition/imbrication of rings resulting from the displacement of a few consecutive rings (figure 4*b*, electronic supplementary material, figures S6 and S7) and their partial dissolution (figure 4*a*, electronic supplementary material, figure S7c,d). The local imbrication of rings in *Corumbella* specimens suggests that these elements were only loosely attached to each other. Although it is unclear whether connective tissues were originally present, these connecting zones were more prone to decay and/or breakage during the pre-burial and post-burial history of the fossil. A similarly loose connection of the rings was previously inferred in *Shaanxilithes* and *Gaojiashania*, two late Ediacaran tubicolous taxa from China [40,41].

More than two superposed wall layers would probably be commonly observed as well if the tubes had a quadrangular cross-section (electronic supplementary material, figure S9b,c). If the perpendicularly oriented walls collapsed outwards, then other faces should be apparent in the fossils, similar to compressed conulariid tubes (e.g. [39]), and compressed artificial square tubes [36–38]. However, when more than two ring series are observed in bedding plane views of *Corumbella*, the extra ring series are much narrower and clearly protrude from below laterally, indicating that they represent partially exposed ring series from the overlain side of the tube (figure 2*a*,*g*,*h*). An outward deformation would also drastically affect the tubes’ width, which is not supported by the normal distribution of the biseriate taphomorph. If the vertically oriented faces collapsed inwards (electronic supplementary material, figure S9b,c), then the transverse and longitudinal sections of *Corumbella’*s tubes should show a superposition of (at least) three to four wall layers. If the walls were bi-layered, this would create a stacking minimum of six to eight superposed ring layers, which does not seem to correspond to the usual two ring layers observed here.

In conclusion, several lines of evidence indicate that the tube of *Corumbella* has an original circular cross-section (i.e. conico-cylindrical shape), as also proposed by Walde *et al*. [23], including (i) the existence of large uniseriate tubes; (ii) the occurrence of both uniseriate and biseriate patterns at apical- and apertural-most portions; (iii) presence of smooth curvilinear rings instead of angular lateral edges; (iv) less-compacted specimens with flattened semicircular shapes; (v) evidence of breakage and limited superposition at the mid-span of the tubes; and (vi) rings crossing the central division. *Corumbella* tubes consisted of a single-layered wall made of ring units, which appeared as two wall layers in transverse and longitudinal sections after compaction of the tube. The rings were originally abutting one another, their occasional imbrication in some specimens resulting from taphonomy. The rings are also elongate ellipsoids in section, with rare crescentic or sigmoidal forms, the latter probably due to deformation at the contact point between ring units. No internal protrusions of the skeleton (e.g. septa, carinae) were observed; previous descriptions of such structures misinterpreted the imprints of the midlines on moulds, which in fact simply evidence the central collapse of the upper and lower sides of the tubes.

### 4.2. Comparisons with other Ediacaran fossils

The reinterpretation of *Corumbella*’s tube as rounded and composed of a series of bordering single-layered rings approximates its morphology to other tubular taxa from the late Ediacaran. In particular, *Corumbella* is strikingly similar to *Costatubus bibendi* and *Costatubus kirsanovi* [16], which suggests that these species are closely related, even possibly congeneric and therefore would be best united within the family Corumbellidae [25]. While awaiting a formal systematic revision of this family, we propose to use the term ‘corumbellomorphs’ in reference to those taxa which share tubes composed of non-imbricating rings and of conico-cylindrical shapes. The ring units also show a general ellipsoid or allantoid outlines in longitudinal sections of the tubes. However, *Corumbella* rings in section have usually more straight lateral margins, while *Costatubus bibendi* has more convex ones. *Corumbella werneri* also differs from *Costatubus bibendi* by the more limited height of its rings (less than or equal to 0.35 mm, instead of up to 1 mm), and these two species can be discriminated from *C. kirsanovi* by the gentler tapering of their tubes and the rather homogeneous height of their rings. In *Costatubus kirsanovi*, the increase in ring width along the tube length is accompanied with an increase in ring height. Differences in tube preservation possibly related to differences in original composition are also worth noting. The tubes of *Corumbella* are preserved as calcite, probably after an originally biomineralized structure (probably aragonite, see [22]). Those of *Costatubus bibendi* are preserved as pyrite, but may also have been biomineralized [16]. The preservation and original composition of the tubes in *Costatubus kirsanovi* have not been investigated.

Corumbellomorphs share with the terminal Ediacaran cloudinomorphs and sinotubulitids the possession of a (probably) biomineralized, multi-element, rounded tube. However, they show no evidence of an original and consistent imbrication of the composing elements (i.e. the rings), even less so their nesting, a diagnostic feature of cloudinomorphs (*sensu* [16]) and sinotubulitids (e.g. [42]), with their ‘funnel-in-funnel’ and ‘cylinder-in-cylinder’ constructions, respectively. This speaks to a radically different mode of tube construction that does not involve a multi-layered structure in corumbellomorphs. Additionally, corumbellomorph tubes are composed of relatively smooth rings, which notably differ from the transversally ridged cylinders of sinotubulitids and the funnel-shaped elements of cloudinomorphs. Future work should investigate whether a corumbellomorph-type construction also occurs in other terminal Ediacaran taxa possessing regularly annulated, non-collared, organic or lightly biomineralized tubes, including *Annulitubus flexuosus* and *Sekwitubulus annulatus* from the Blueflower Formation in Canada [43]; *Convolutubus dargazinensis* from the Kushk Series of Iran [44]; and *Wutubus annularis* from the Dengying Formation in China [45].

Occurrences of tubes with supposed fourfold symmetries that were previously interpreted as *Corumbella* should be considered as distinct taxa (figs. 4–6 in [46] and fig. 4d in [47]). Fossils of *Corumbella* found in Paraguay [32,48] and central Brazil [49], which also lack midlines and polyhedral shapes are here considered valid occurrences of the genus. Recently reported putative *Corumbella* remains from Namibia [50] require further investigation to confirm their taxonomic assignment to this genus or other corumbellomorphs. *Corumbella* was also reported from Iran [44,51,52], and while some of the illustrated specimens share some resemblance to corumbellomorphs, others may represent a new, apparently organic, tubular taxon.

## 5. Conclusions

*Corumbella* is reinterpreted as originally having a conico-cylindrical tube with a single-layered wall and no internal septa. Previously hypothesized features of these Ediacaran shelly fossils, such as a conico-pyramidal shape, a complex imbrication of their composing elements and a multi-layering of their walls, result from a misinterpretation of the impact of taphonomy—especially compaction—on the original morphology of these multi-element skeletons. Critically, we demonstrate that the midline—a key anatomical character supporting the hypothesis of fourfold symmetry in *Corumbella*’s tube—is actually a compaction crack similar to those produced experimentally in organic and biomineralized annelid tubes.

The tubes of *Corumbella* and its close relative *Costatubus* are composed of loosely connected, non-imbricated rings. This ‘barrel-on-barrel’ organization significantly differs from the nesting of complex skeletal elements characterizing cloudinomorphs and sinotubulitids, although it may occur in other terminal Ediacaran tubicolous taxa. Our new reconstruction of *Corumbella*, especially the absence of internal septa and fourfold symmetry, challenges the long-standing hypothesis of a close phylogenetic relationship with scyphozoan cnidarians, especially coronates and conulariids [21,24,25,27]. While this study provides valuable insights into the skeletal anatomy of this ancient animal, it does not resolve the placement of this Ediacaran taxon within the animal tree of life.

## Data Availability

All data is available in the manuscript and its supplementary material [53].

## References

[1] Wood R, Ivantsov AY, Zhuravlev AY. 2017 First macrobiota biomineralization was environmentally triggered. Proc. R. Soc. B **284**, 20170059. (10.1098/rspb.2017.0059) PMC5378091 28356454

[2] Becker-Kerber B, Pacheco MLAF, Rudnitzki ID, Galante D, Rodrigues F, Leme J de M. 2017 Ecological interactions in Cloudina from the Ediacaran of Brazil: implications for the rise of animal biomineralization. Sci. Rep. **7**, 5482. (10.1038/s41598-017-05753-8) 28710440 PMC5511220

[3] Yang B, Warren LV, Steiner M, Smith EF, Liu P. 2022 Taxonomic revision of Ediacaran tubular fossils: Cloudina, Sinotubulites and Conotubus. J. Paleontol. **96**, 256–273. (10.1017/jpa.2021.95)

[4] Becker-Kerber B, da Silva FR, Amorim KB, Pacheco MLAF, Leme J de M. 2019 Putting the cart before the horse: an example of how the lack of taphonomical approaches can mislead paleobiological inferences for the late Ediacaran. Precambrian Res. **332**, 105385. (10.1016/j.precamres.2019.105385)

[5] Zhuravlev Ay, Wood RA, Penny AM. 2015 Ediacaran skeletal metazoan interpreted as a lophophorate. Proc. R. Soc. B. **282**, 1–10. (10.1098/rspb.2015.1860) PMC4650157 26538593

[6] Schiffbauer JD, Selly T, Jacquet SM, Merz RA, Nelson LL, Strange MA, Cai Y, Smith EF. 2020 Discovery of bilaterian-type through-guts in cloudinomorphs from the terminal Ediacaran period. Nat. Commun. **11**, 1–12. (10.1038/s41467-019-13882-z) 31924764 PMC6954273

[7] Schiffbauer JD, Huntley JW, O’Neil GR, Darroch SAF, Laflamme M, Cai Y. 2016 The latest Ediacaran wormworld fauna: setting the ecological stage for the Cambrian explosion. GSA Today **26**, 4–11. (10.1130/GSATG265A.1)

[8] Yang B, Steiner M, Zhu M, Li G, Liu J, Liu P. 2016 Transitional Ediacaran–Cambrian small skeletal fossil assemblages from South China and Kazakhstan: implications for chronostratigraphy and metazoan evolution. Precambrian Res. **285**, 202–215. (10.1016/j.precamres.2016.09.016)

[9] Vinn O, Nanglu K, Wilson MA, Isakar M, Toom U. 2025 Ediacaran-type non-mineralized tube-dwelling organisms persisted into the early Cambrian (Terreneuvian) in Baltica. Gondwana Res. **137**, 29–35. (10.1016/j.gr.2024.09.009)

[10] Cai Y, Xiao S, Li G, Hua H. 2019 Diverse biomineralizing animals in the terminal Ediacaran Period herald the Cambrian explosion. Geology **47**, 380–384. (10.1130/G45949.1)

[11] Waggoner B. 2003 The Ediacaran biotas in space and time. Integr. Comp. Biol. **43**, 104–113. (10.1093/icb/43.1.104) 21680415

[12] Muscente AD *et al*. 2019 Ediacaran biozones identified with network analysis provide evidence for pulsed extinctions of early complex life. Nat. Commun. **10**, 1–15. (10.1038/s41467-019-08837-3) 30796215 PMC6384941

[13] Germs GJB. 1972 New shelly fossils from Cama Group, South West Africa. Am. J. Sci. **272**, 752–761. (10.2475/ajs.272.8.752)

[14] Cai Y, Xiao S, Hua H, Yuan X. 2015 New material of the biomineralizing tubular fossil Sinotubulites from the late Ediacaran Dengying Formation, South China. Precambrian Res. **261**, 12–24. (10.1016/j.precamres.2015.02.002)

[15] Cai Y, Cortijo I, Schiffbauer JD, Hua H. 2017 Taxonomy of the late Ediacaran index fossil Cloudina and a new similar taxon from South China. Precambrian Res. **298**, 146–156. (10.1016/j.precamres.2017.05.016)

[16] Selly T *et al*. 2020 A new cloudinid fossil assemblage from the terminal Ediacaran of Nevada, USA. J. Syst. Palaeontol. **18**, 357–379. (10.1080/14772019.2019.1623333)

[17] Cai Y, Schiffbauer JD, Hua H, Xiao S. 2011 Morphology and paleoecology of the late Ediacaran tubular fossil Conotubus hemiannulatus from the Gaojiashan Lagerstätte of southern Shaanxi province, South China. Precambrian Res. **191**, 46–57. (10.1016/j.precamres.2011.09.002)

[18] Yang B, Steiner M, Schiffbauer JD, Selly T, Wu X, Zhang C, Liu P. 2020 Ultrastructure of Ediacaran cloudinids suggests diverse taphonomic histories and affinities with non-biomineralized annelids. Sci. Rep. **10**, 535. (10.1038/s41598-019-56317-x) 31953458 PMC6968996

[19] Warren LV *et al*. 2024 The Ediacaran paleontological record in South America. Earth Sci. Rev. **258**, 104915. (10.1016/j.earscirev.2024.104915)

[20] Warren LV, Pacheco MLAF, Fairchild TR, Simões MG, Riccomini C, Boggiani PC, Cáceres AA. 2012 The dawn of animal skeletogenesis: ultrastructural analysis of the Ediacaran metazoan Corumbella werneri. Geology **40**, 691–694. (10.1130/g33005.1)

[21] Pacheco MLAF *et al*. 2015 Insights into the skeletonization, lifestyle, and affinity of the unusual Ediacaran fossil Corumbella. PLoS One **10**, 1–19. e0114219. (10.1371/journal.pone.0114219) PMC4379158 25822998

[22] Osés GL, Wood R, Romero GR, Prado GMEM, Bidola P, Herzen J, Pfeiffer F, Stampar SN, Pacheco MLAF. 2022 Ediacaran Corumbella has a cataphract calcareous skeleton with controlled biomineralization. iScience **25**, 105676. (10.1016/j.isci.2022.105676) 36561886 PMC9763863

[23] Walde DHG, Weber B, Erdtmann BD, Steiner M. 2019 Taphonomy of Corumbella werneri from the Ediacaran of Brazil: sinotubulitid tube or conulariid test? Alcheringa **43**, 335–350. (10.1080/03115518.2019.1615551)

[24] Babcock LE, Grunow AM, Sadowski GR, Leslie SA. 2005 Corumbella, an Ediacaran-grade organism from the late Neoproterozoic of Brazil. Palaeogeogr. Palaeoclimatol. Palaeoecol. **220**, 7–18. (10.1016/j.palaeo.2003.01.001)

[25] Hahn G, Hahn R, Leonardos OH, Pflug HD, Walde DHG. 1982 Körperlich erhaltene scyphozoen-reste aus dem Jungpräkambrium Brasiliens. Geol. Palaeontol. **16**, 1–18.

[26] Dunn FS, Kenchington CG, Parry LA, Clark JW, Kendall RS, Wilby PR. 2022 A crown-group cnidarian from the Ediacaran of Charnwood Forest, UK. Nat. Ecol. Evol. **6**, 1095–1104. (10.1038/s41559-022-01807-x) 35879540 PMC9349040

[27] Van Iten H, Marques AC, Leme J de M, Pacheco M, Simões MG. 2014 Origin and early diversification of the phylum Cnidaria Verrill: major developments in the analysis of the taxon’s Proterozoic–Cambrian history. Palaeontology **57**, 677–690. (10.1111/pala.12116)

[28] Van Iten H, Leme JM, Pacheco M, Simões MG, Fairchild TR, Rodrigues F, Galante D, Boggiani PC, Marques AC. 2016 Origin and early diversification of phylum Cnidaria: key macrofossils from the Ediacaran system of North and South America. In The Cnidaria, past, present and future: the world of medusa and her sisters, pp. 31–40. Cham, Switzerland: Springer International Publishing. (10.1007/978-3-319-31305-4_3)

[29] Pinto A, Borin G, Carlos B, Bernardi ML, Sarmento MF, Peixinho AZ, Spina TV, Miqueles EX. 2022 Annotat3D: a modern web application for interactive segmentation of volumetric images at sirius/LNLS. Synchrotron Radiat. News **35**, 36–43. (10.1080/08940886.2022.2112501)

[30] Pacheco M, Leme J, Machado A. 2011 Taphonomic analysis and geometric modelling for the reconstitution of the Ediacaran metazoan Corumbella werneri Hahn et al. 1982 (Tamengo Formation Corumbá Basin, Brazil). J. Taphon. **9**, 269–283.

[31] Osés GL *et al*. 2025 Clastic sedimentary record impacted by carbonate bioclasts in the late Ediacaran. Geol. Mag. **162**, e2. (10.1017/S0016756824000335)

[32] Warren LV, Fairchild TR, Gaucher C, Boggiani PC, Poiré DG, Anelli LE, Inchausti JCG. 2011 Corumbella and in situ Cloudina in association with thrombolites in the Ediacaran Itapucumi Group, Paraguay. Terra Nov. **23**, 382–389. (10.1111/j.1365-3121.2011.01023.x)

[33] Li S, Guo X, Li Q, Ruan D, Sun G. 2020 On lateral compression of circular aluminum, CFRP and GFRP tubes. Compos. Struct. **232**, 111534. (10.1016/j.compstruct.2019.111534)

[34] Skovsted CB, Peel JS. 2011 Hyolithellus in life position from the Lower Cambrian of North Greenland. J. Paleontol. **85**, 37–47. (10.1666/10-065.1)

[35] Zhu M yan, Van Iten H, Cox RS, Zhao Y long, Erdtmann BD. 2000 Occurrence of Byronia Matthew and Sphenothallus Hall in the Lower Cambrian of China. Paläont. Z. **74**, 227–238. (10.1007/BF02988098)

[36] Gupta NK, Ray P. 1998 Collapse of thin-walled empty and filled square tubes under lateral loading between rigid plates. Int. J. Crashworthiness **3**, 265–285. (10.1533/cras.1998.0075)

[37] Gupta NK, Sekhon GS, Gupta PK. 2001 A study of lateral collapse of square and rectangular metallic tubes. Thin Walled Struct. **39**, 745–772. (10.1016/s0263-8231(01)00033-7)

[38] Tran TN, Ton TNT. 2016 Lateral crushing behaviour and theoretical prediction of thin-walled rectangular and square tubes. Compos. Struct. **154**, 374–384. (10.1016/j.compstruct.2016.07.068)

[39] Van Iten H, Gutiérrez-Marco JC, Cournoyer ME. 2022 Unusual assemblage of conulariids (Cnidaria, Scyphozoa) from the Taddrist Formation (Middle Ordovician, Darriwilian) of southern Morocco. J. Paleontol. **96**, 803–813. (10.1017/jpa.2022.6)

[40] Cai Y, Hua H, Xiao S, Schiffbauer JD, Li P. 2010 Biostratinomy of the late Ediacaran pyritized Gaojiashan Lagerstatte from southern Shaanxi, South China: importance of event deposits. Palaios **25**, 487–506. (10.2110/palo.2009.p09-133r)

[41] Meyer M, Schiffbauer JD, Xiao S, Cai Y, Hua H. 2012 Taphonomy of the upper Ediacaran enigmatic ribbonlike fossil Shaanxilithes. Palaios **27**, 354–372. (10.2110/palo.2011.p11-098r)

[42] Chen Z, Bengtson S, Zhou CM, Hua H, Yue Z. 2008 Tube structure and original composition of Sinotubulites: shelly fossils from the late Neoproterozoic in southern Shaanxi, China. Lethaia **41**, 37–45. (10.1111/j.1502-3931.2007.00040.x)

[43] Carbone CA, Narbonne GM, Macdonald FA, Boag TH. 2015 New Ediacaran fossils from the uppermost Blueflower Formation, northwest Canada: disentangling biostratigraphy and paleoecology. J. Paleontol. **89**, 281–291. (10.1017/jpa.2014.25)

[44] Vaziri SH, Majidifard MR, Darroch SAF, Laflamme M. 2021 Ediacaran diversity and paleoecology from central Iran. J. Paleontol. **95**, 236–251. (10.1017/jpa.2020.88)

[45] Chen Z, Zhou C, Xiao S, Wang W, Guan C, Hua H, Yuan X. 2014 New Ediacara fossils preserved in marine limestone and their ecological implications. Sci. Rep. **4**, 1–10. (10.1038/srep04180) PMC3933909 24566959

[46] Hagadorn JW, Waggoner B. 2000 Ediacaran fossils from the southwestern Great Basin, United States. J. Paleontol. **74**, 349–359. (10.1017/s0022336000031553)

[47] Smith EF, Nelson LL, Tweedt SM, Zeng H, Workman JB. 2017 A cosmopolitan late Ediacaran biotic assemblage: new fossils from Nevada and Namibia support a global biostratigraphic link. Proc. R. Soc. B **284**, 20170934. (10.1098/rspb.2017.0934) PMC5524506 28701565

[48] Warren LV, Quaglio F, Simões MG, Gaucher C, Riccomini C, Poiré DG, Freitas BT, Boggiani PC, Sial AN. 2017 Cloudina-Corumbella-Namacalathus association from the Itapucumi Group, Paraguay: increasing ecosystem complexity and tiering at the end of the Ediacaran. Precambrian Res. **298**, 79–87. (10.1016/j.precamres.2017.05.003)

[49] Warren LV, Quaglio F, Riccomini C, Simões MG, Poiré DG, Strikis NM, Anelli LE, Strikis PC. 2014 The puzzle assembled: Ediacaran guide fossil Cloudina reveals an old proto-Gondwana seaway. Geology **42**, 391–394. (10.1130/g35304.1)

[50] Turk KA, Maloney KM, Laflamme M, Darroch SAF. 2022 Paleontology and ichnology of the late Ediacaran Nasep–Huns transition (Nama Group, southern Namibia). J. Paleontol. **96**, 753–769. (10.1017/jpa.2022.31)

[51] Vickers-Rich P, Soleimani S, Farjandi F, Zand M, Linnemann U, Hofmann M, Wilson SA, Cas R, Rich TH. 2018 A preliminary report on new Ediacaran fossils from Iran. Alcheringa **42**, 230–243. (10.1080/03115518.2017.1384061)

[52] Vaziri SH, Majidifard MR, Laflamme M. 2018 Diverse assemblage of Ediacaran fossils from central Iran. Sci. Rep. **8**, 1–7. (10.1038/s41598-018-23442-y) 29567986 PMC5864923

[53] Becker-Kerber B, Ortega-Hernández J, Schiffbauer J, Lerosey-Aubril R, Warren LV, Simões MG *et al*. 2025. Rebuilding Earth’s First Skeletal Animals: The Original Morphology of Corumbella (Ediacaran, Brazil). FigShare. (10.6084/m9.figshare.c.7817036) PMC12092125 40400519

